# Supplementary material for: Multiethnic Investigation of Risk and Immune Determinants of COVID-19 Outcomes
Source: Front Cell Infect Microbiol. 2022 Jul 22;12:933190. doi: 10.3389/fcimb.2022.933190 (PMC9355800; doi:10.3389/fcimb.2022.933190)
Supplement: Supplementary file 1 [file DataSheet_1.zip › Supplemental Table 1.DOCX]

Supplemental Table 1: Baseline demographic and clinical characteristics of patients presenting to the emergency department, by hospitalization status.

|  | Admitted  (N=3,086) | Not Admitted  (N=1,911) | P-value |
| --- | --- | --- | --- |
| Age (yrs) | 66 (56 - 77) | 50 (36 - 62) | <0.001 |
| Asian | 144 (4.8%) | 86 (4.6%) | 0.781 |
| Hispanic | 892 (29.7%) | 495 (26.4%) | 0.014 |
| Non-Hispanic Black | 825 (27.4%) | 635 (33.9%) | <0.001 |
| Non-Hispanic White | 689 (22.9%) | 397 (21.2%) | 0.168 |
| Other | 458 (15.2%) | 262 (14%) | 0.245 |
| Current smoker | 113 (4.7%) | 78 (5.5%) | 0.282 |
| Former smoker | 658 (27.4%) | 204 (14.4%) | <0.001 |
| Never smoker | 1629 (67.9%) | 1133 (80.1%) | <0.001 |
| Hypertension | 1096 (35.5%) | 258 (13.5%) | <0.001 |
| Diabetes | 741 (24%) | 149 (7.8%) | <0.001 |
| Coronary artery disease | 395 (12.8%) | 78 (4.1%) | <0.001 |
| Heart failure | 218 (7.1%) | 36 (1.9%) | <0.001 |
| Atrial fibrillation | 201 (6.5%) | 36 (1.9%) | <0.001 |
| Chronic kidney disease | 368 (11.9%) | 59 (3.1%) | <0.001 |
| COPD/asthma | 265 (8.6%) | 86 (4.5%) | <0.001 |
| Obesity | 250 (8.1%) | 91 (4.8%) | <0.001 |
| Cancer | 205 (6.6%) | 59 (3.1%) | <0.001 |
| Chronic liver disease | 83 (2.7%) | 28 (1.5%) | 0.004 |
| Obstructive sleep apnea | 65 (2.1%) | 19 (1%) | 0.003 |
| HIV | 56 (1.8%) | 21 (1.1%) | 0.058 |
| Temperature (°F) | 98.9 (98.2 - 100.2) | 98.6 (97.9 - 99.7) | <0.001 |
| Heart rate (bpm) | 97 (84 - 110) | 92 (81 - 104) | <0.001 |
| Systolic blood pressure (mmHg) | 129 (115 - 146) | 131 (119 - 144) | 0.02 |
| Respiratory rate (bpm) | 20 (18 - 22) | 18 (17 - 20) | <0.001 |
| Oxygen saturation (%) | 95 (91 - 97) | 98 (96 - 99) | <0.001 |
| Oxygen sat. <92% | 775 (25.1%) | 96 (5%) | <0.001 |

Values represent count (%) or median (IQR) for categorical and continuous variables, respectively.
